# Intermittent Operation of CO_2_ Electrolyzers at Industrially Relevant Current Densities

**DOI:** 10.1021/acsenergylett.2c00923

**Published:** 2022-05-04

**Authors:** Angelika A. Samu, Attila Kormányos, Egon Kecsenovity, Norbert Szilágyi, Balázs Endrődi, Csaba Janáky

**Affiliations:** † Department of Physical Chemistry and Materials Science, University of Szeged, Rerrich Square 1, Szeged H-6720, Hungary; ‡ eChemicles Zrt, Alsó Kikötő sor 11, Szeged H-6726, Hungary

## Abstract

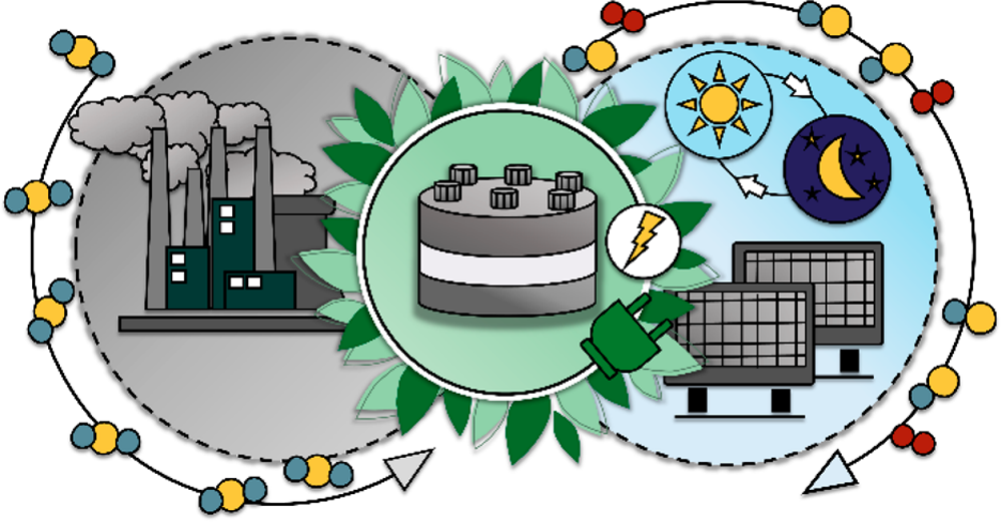

We demonstrate the dynamic operation of CO_2_ electrolyzer cells, with a power input mimicking the output of a solar photovoltaic power plant. The zero-gap design ensured efficient intermittent operation for a week, while avoiding significant performance loss.

In the future electrical energy grid, an increasing fluctuation of the power load is expected because of the growing amount of intermittent renewable energy in the electricity mix.^1^ Beyond the challenges this poses for the electricity infrastructure, it also results in a massive fluctuation in electricity prices. Electrochemical power-to-gas and power-to-liquid technologies are promising chemical energy conversion approaches to be coupled with intermittent renewable energy sources to balance the grid while utilizing cheap excess energy at peak times.^2^ Electrochemical hydrogen generation methods (such as proton exchange membrane (PEM) water electrolyzers) are well known for their dynamic response to external electrical power load; therefore, they can be directly coupled to different renewable energy sources, such as windmills or solar photovoltaic (PV) power plants.

Direct CO_2_ electrolyzers operating at industrially relevant current densities^3−5^ can, in principle, offer similar opportunities, although no experimental evidence has been demonstrated on this matter yet. Such verification would be very important, considering the notable differences compared to water electrolyzers (e.g., CO_2_ gas feed at the cathode, the use of soft cathode gas diffusion electrode (GDE), etc.). Specifically, fluctuations in the local pressure, due to the rapid increase/decrease in the reaction rate, might cause flooding in the cathode GDE, which can be detrimental for the stability of the electrolyzer cell. A few studies targeted dynamic operation, but they were limited to on/off switching cycles in short measurements and some simple variation of the potential/current to regenerate the Cu catalyst and/or avoid precipitate formation in the electrolyzer cells.^6−10^ Low-temperature CO_2_ electrolyzers are particularly promising for dynamic operation, while high-temperature systems are challenged by their thermal management (especially large-scale systems).^11^ In this Energy Express, we compare the dynamic operation of two low-temperature CO_2_ electrolyzer cells: a membrane-less microfluidic and a zero-gap cell, to provide *the first experimental demonstration* of an electrolyzer cell absorbing a power load mimicking a PV power plant, generating CO for a whole week.

First, we have developed an environment (Figure S1) for the autonomous testing of CO_2_ electrolyzer cells (Figure S2) in the CO_2_-to-CO conversion process (Figure S3). As the first step, we defined a dynamic current control protocol to test the response of zero-gap electrolyzer cells in terms of cell voltage and CO/H_2_ partial current densities (*j*_CO_ and *j*_H_2__). The process consisted of alternating 8 h periods of constant current and dynamically changing current operation (see Figure S4). We decided to control the current density of the cell because proper power electronics are available for both DC/AC/DC and DC/DC conversions, as demonstrated on the example of PEM water electrolyzers.^12^ Comparison with constant current operation (Figure S5) shows that the stability (i.e., cell voltage and product distribution) is not affected by the dynamically changing current pattern. In fact, one may even consider some possible beneficial effect of the current fluctuation, such as the release of entrapped gas bubbles.

Subsequently, we applied a protocol which mimics the power output of a solar PV power plant in Germany, where data was available with 1 min resolution.^13^ Total current densities were linearly scaled with the PV power output, with a maximum of 437.5 mA cm^–2^. The cell power load profile slightly differs from the PV power output (due to the varying cell voltage), and our current control protocol enhances the fluctuations in the power (see the comparison in Figure S6). As shown in Figure 1B–D, *j*_CO_ follows the shape of the total current curve (FE_H_2__ remains below 15%). The cell voltage curve also resembles that of the power input (Figure 1B), although with a ca. 2.15 V threshold value, the onset voltage of CO_2_ electrolysis in the presented cell. Clearly, the electrolyzer performance follows the dynamic power load with no detectable delay (Figure 1C), and a total FE of 92–95% was detected (Figure S7). To assess the long-term stability of the cell under these conditions, we ran the electrolyzer for a week. As seen in Figure 1D, the CO partial current density closely follows the input power pattern, indicating no significant degradation issue.

**Figure 1:**
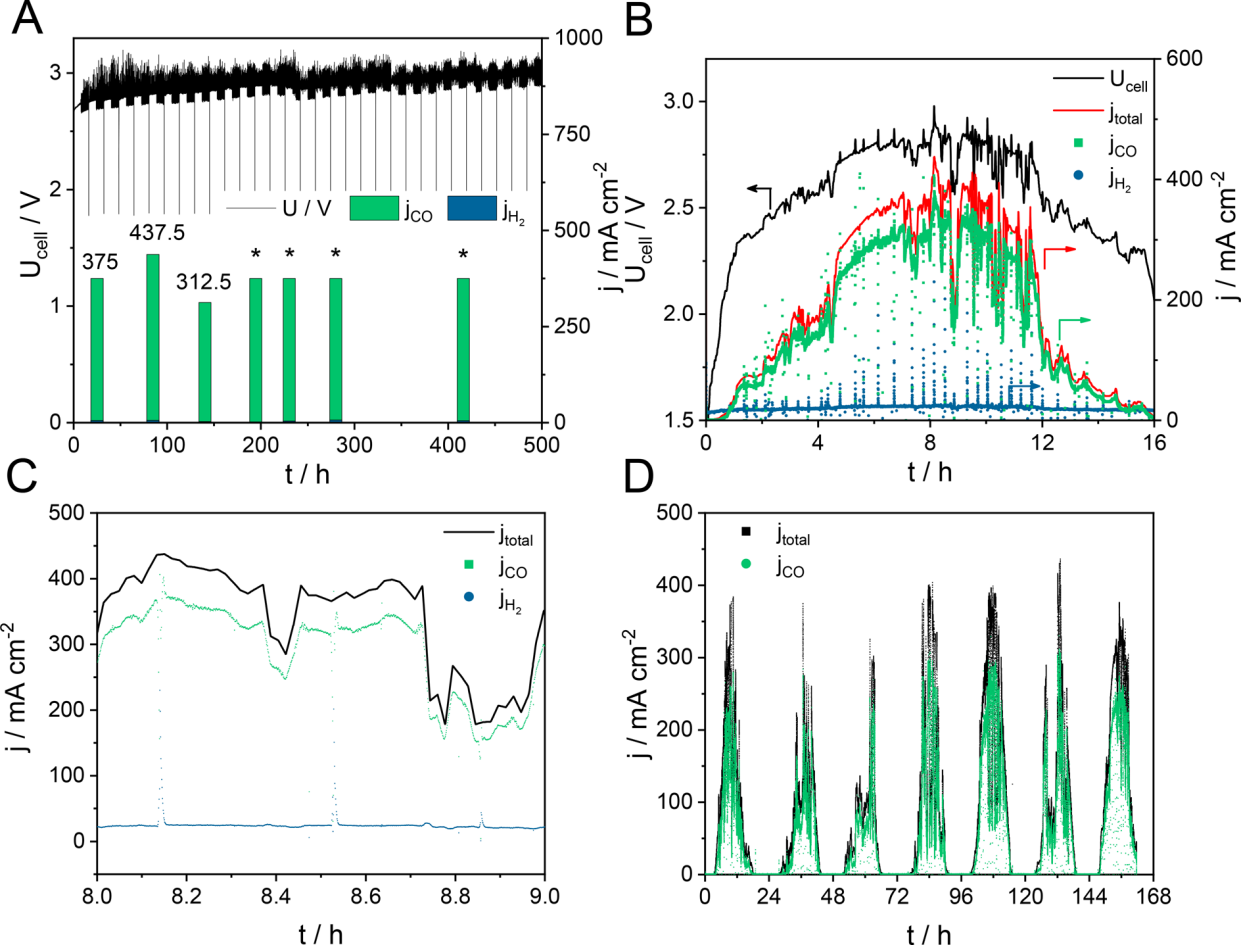
(A) Cell voltage and product distribution during electrolysis according to the current protocol shown in Figure S4. The asterisks mark partial current density values obtained at *j* = 375 mA cm^–2^ total current density, while the analysis at 90 and 140 h was performed at 437.5 and 312.5 mA cm^–2^, respectively. Cell voltage and (partial) current density during electrolysis at a continuously changing current density, following the power profile of a solar PV plant (B–D) for different time periods.

To check whether this capability is a unique feature of zero-gap cells or can be stretched to microfluidic devices, we extended our studies to this latter group. Figure S8 illustrates stable operation at fixed current density, but the cell gradually gets flooded once the current density is varied. The rapidly changing gas evolution rate (O_2_, CO, and H_2_) can induce rapid fluctuation in the local pressure. As the gas/solution can penetrate into the carbon paper even at low differential pressures,^14,15^ it is not surprising that the GDE gets flooded.

Overall, we demonstrated that the studied zero-gap cell can properly function under intermittent operational conditions, while its microfluidic counterpart suffers from rapid flooding under such circumstances. Further efforts are ongoing to elucidate the effect of dynamic power load on multicell stacks and multistack systems (together with uncovering failure mechanisms), because these insights are very important in designing electrochemical cell configurations and energy conversion systems to scale-up this promising technology.

## Supporting Information Available

Experimental methods, including electrode preparation, cell configurations, and electrochemical measurements, with Figures S1–S8 (PDF)
