# Supplementary material for: Intermittent Operation of CO2 Electrolyzers at Industrially Relevant Current Densities
Source: ACS Energy Lett. 2022 May 4;7(5):1859–61. doi: 10.1021/acsenergylett.2c00923 (PMC9112675; doi:10.1021/acsenergylett.2c00923)
Supplement: Supplementary file 1 — nz2c00923_si_001.pdf [file nz2c00923_si_001.pdf]

# – Supporting Information –

Intermittent Operation of CO_2 Electrolyzers at Industrially Relevant Current Densities

Angelika A. Samu[1,2], Attila Kormányos[1], Egon Kecsenovity[1], Norbert Szilágyi[1], Balázs Endrődi[1] and Csaba Janáky[1,2]*

[1]Department of Physical Chemistry and Materials Science, University of Szeged,

Rerrich Square 1, Szeged, H-6720, Hungary

[2]eChemicles Zrt, Alsó Kikötő sor 11, Szeged, H-6726, Hungary

AUTHOR INFORMATION

Corresponding Author

*janaky@chem.u-szeged.hu (C. Janáky)

Experimental details

Electrode preparation and membrane pretreatment

In the experiments using a zero-gap electrolyzer cell, the catalyst-coated electrodes were separated with a PTFE supported anion exchange membrane (Sustainion® X37-50 grade T, Dioxide Materials). A Ag nanoparticle coated gas diffusion electrode was used as the cathode and an Iridium Black coated porous Ti frit as the anode.

To prepare the cathode gas diffusion electrode, Ag nanoparticles ($d_{avg} < 100$ nm, Sigma-Aldrich) were dispersed in a 1:1 isopropanol-water solvent mixture at a concentration of 25 mg cm^{-3}. The dispersion also contained 15 m/m% Sustainion® XC-2 alkaline ionomer (Dioxide Materials), with regards to the total mass of the catalyst and the ionomer together. The 20 mg cm^{-3} dispersion of Ir nanoparticles (Fuel-Cell Store) was formed in an identical solvent mixture and alkaline ionomer concentration, using Sustainion® XB-7 (Dioxide Materials) ionomer. The Ir dispersion was homogenized in an ultrasonic bath for 20 minutes (keeping the bath temperature below 35 °C), while a high-power immersion sonotrode (Hielscher UP200ST) was used to disperse the Ag nanoparticles.

The silver dispersion was spray-coated with a hand-held airbrush on Freudenberg H23C6 gas diffusion layer (GDL, purchased from QuinTech) on a preheated hotplate at 100 °C. The anode catalyst was spray-coated similarly, on a porous titanium frit for the measurements in zero-gap electrolyzer cells, and on Freudenberg H23C6 GDL for the experiments in microfluidic electrolyzer cells. The cathode and the anode catalyst loadings were 1.0 ± 0.1 mg cm^{-2}. We immersed the cathode gas diffusion electrode in 0.05 M CsOH (the catalyst layer facing the liquid surface) for at least 15 minutes before inserted in the electrolyzer cell to convert the ionomer to hydroxide form.

The anion exchange membrane was activated for at least 24 hours by immersing it in 1 M CsOH. Subsequently it was cut into shape using a surgical blade, washed with ample

amount of deionized (DI) water, and inserted in the electrolyzer cell in its fully hydrated form. The thickness of the employed Sustainion anion exchange membrane was about 60 μm.

To prepare the GDEs employed in the microfluidic electrolyzer cell Ag nanoparticles (d_{avg} < 100 nm, Sigma-Aldrich) were dispersed in a 1:1 isopropanol-water solvent mixture at a concentration of 25 mg cm^{-3}. The dispersion also contained 15 m/m% Nafion® ionomer (Fuel-Cell Store), with regards to the total mass of the catalyst and the ionomer together. The 20 mg cm^{-3} dispersion of Ir nanoparticles (Fuel-Cell Store) was formed in an identical solvent and Nafion® mixture using an identical ionomer concentration as for the Ag dispersion. The Ir dispersion was homogenized in an ultrasonic bath for 20 minutes (keeping the bath temperature below 35 °C), while a high-power immersion sonotrode (Hielscher UP200ST) was used to disperse the Ag nanoparticles. GDEs were prepared in a similar manner (type of carbon paper, catalyst loading, spray-coating parameters, etc.) as in the case of the zero-gap cell except that the Ir dispersion was spray-coated also on a Freudenberg H23C6 carbon paper GDL, instead of a titanium frit. The reason behind this, is that in the microfluidic cell, the liquid and gas phases must be separated at the anode side, similarly to how it is done at the cathode. Using a non-hydrophobic substrate (e.g., Ti frit), the electrolyte solution would penetrate through that instead of flowing through the microchannel. On the other hand, in zero-gap cells, the solution must penetrate through the substrate to reach the catalyst surface, hence hydrophobic carbon papers are not suitable porous electrodes here.

Test framework and electrochemical measurements

For our measurements, we developed a test station that allows us to operate four electrolyzer cells simultaneously, in a fully automated manner. Using multiple channels allows us to obtain information on the effect of several experimental parameters at the same time. This test station was based on our previously reported experimental set-up.[1] Briefly, the CO_2 feed rate was

controlled with a Bronkhorst MASS-STREAM D-6321 type mass-flow controller (MFC), while a Bronkhorst MASS-STREAM D-6310 type mass flow meter (MFM) was used to measure the flow rate of the gas outlet. The gas flow rate was normalized with the surface area of the cell (hence the units of cm^3 cm^{-2} min^{-1}). The CO_2 gas was passed through a temperature-controlled humidifier before entering the cell. The level of the DI water in the gas humidifier was kept constant by periodically refilling it. The pressure of the CO_2 gas in the electrolyzer was measured using a Festo digital pressure transmitter. The anolyte was circulated in the anode compartment at a rate of 80 cm^3 min^{-1} using a peristaltic pump. The short-term electrochemical measurements presented in this paper were performed using a Biologic VMP- 300 type instrument, equipped with high current (up to 10 A) booster, while a Rohde & Schwarz HMP4040 power supply was applied for the long-term operation.

We built a custom-designed hardware and software for the long-term operation test station. An Atmel Atmega2560 microcontroller was used for analog sensors (such as thermistors) with its built-in A/D converter and providing that data through a serial interface. It was also used for temperature control. A data acquisition software was made for a PC with a user-friendly interface, written in LabVIEW. This software was responsible for controlling the power supply's output, the heaters (gas humidifier, gas line, anolyte), the thermal water separator, the automatic water separator emptying valve and the MFC in the setup, and for synchronizing the sampling on all instruments. We used software timing for sampling and buffers to be able to merge the data coming from different instruments with different latency into a common log file. Therefore, there can be a minimal time lag in sampling between the instruments, but it should be always less than one second. No filtering was used, all logged data are momentary.

The measurements were performed in a two-electrode set-up, and the total cell voltage (U_{cell}) is given as the voltage difference between the anode and the cathode (hence the positive

values). The absolute value of the current/current density is shown (positive values) in all figures. No IR correction was applied on the voltage values presented throughout the manuscript. The experimental conditions for all experiments with zero gap cells were: 0.05 M $CsHCO_3$ anolyte, recirculated at a rate of ca. 80 cm^3 min^{-1}, 12.5 cm^3 cm^{-2} min^{-1} cathodic CO_2 feed, humidified at 60 °C, T = 60 ± 1 °C cell cathode temperature.

The composition of the cathode product stream during the short electrochemical measurements was analyzed using a Shimadzu Nexis-GC-2030 type instrument, equipped with a barrier discharge ionization (BID) detector. A Restek ShinCarbon ST column was employed for the separation, with 6.0 grade helium carrier gas. An automatized six-port valve was used to take samples at regular time intervals. Fort the long-term operation we used an online infrared gas analyzer (Gasboard-3100, customized for CO_2-CO-H_2 mixtures, Hubei Cubic-Ruiyi), which allowed a real-time determination of the product composition. The gas analyzer was calibrated prior to use, and a total Faradaic efficiency over 90 % was obtained in all data points. The timescale of the gas analysis was always shifted by 50 s, a delay caused by the volume of the test system shown in Fig. S1. The formation and anodic oxidation of formate cannot be fully excluded, but no liquid products were detected. The constant loss is therefore probably related to the uncertainty in product quantification.

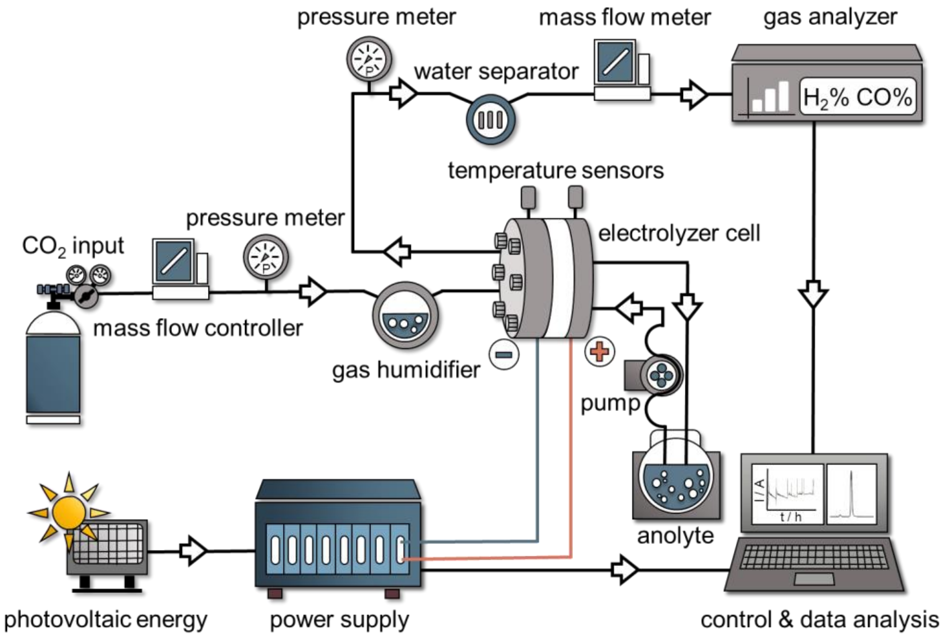

Figure S1. Schematic diagram of the test environment applied for the electrolysis experiments with the zero-gap CO_2 electrolyzer cells.

Electrochemical measurements employing the microfluidic electrolyzer cell were conducted using a different setup, based on a Biologic VMP- 300 type potentiostat/galvanostat. The measurements were conducted in a two-electrode set-up, and the total cell voltage is given as the voltage difference between the anode and the cathode (hence the positive values). No IR correction was applied on the voltage values presented throughout the manuscript. 1 M CsOH was used as the electrolyte, and it was introduced in the cell using a syringe pump (KF Technology model 300) applying 0.5 cm^3 min^{-1} flow rate. The difference in the concentration of the applied electrolyte solution as compared to the measurements with the zero-gap cells (where it was 0.1 M) is to reduce the solution resistance in the microfluidic electrolyzer cell. CO_2 feed rate was controlled at 20 cm^3 cm^{-2} min^{-1} with a Bronkhorst F-201CV type mass-flow controller (MFC), while an Agilent ADM flow meter was used to measure the flow rate of the gas outlet. The composition of the cathode product stream was periodically analyzed with the same GC instrument as described above. Continuous qualification and quantification of the product stream was performed using an m/z analyzer (SRS UGA 200) equipped with an

atmospheric sampling capillary. Pressure variations in the liquid stream was monitored using a Festo digital pressure transmitter (SPTW-B2R-G14-VD-M12) and a DataQ DI-2108 data acquisition unit. The transmitter was connected to the outlet of the electrolyzer cell via a T-connector.

Electrolyzer cell assembly

A custom-designed direct gas feed, zero-gap electrolyzer cell was used for the experiments. The cell components were made of high-quality stainless steel (SS 316Ti). It consists of an anode current collector on which an anolyte flow pattern was formed, a porous Ti frit with catalyst layer on its side in direct contact with the AEM. In addition, a cathode current collector on which the GDE is placed with its catalyst layer facing the membrane. The gas flow channels were formed in the cathode current collectors. Importantly, a CO_2 gas inlet was formed in the center of the circular cathode current collector, while the outlet channel is on the perimeter. During the experiments, the anolyte was recirculated through the anode side and humidified CO_2 was fed to the cathode side. Six bolt screws were used to assemble the cell, gradually applying a torque of 3 Nm. The active surface is circular, of diameter 3.2 cm, resulting in a geometrical surface of area, A = 8 cm^2.

For comparing the effect of the dynamic electrolysis conditions on the stability of different electrolyzer cells, we constructed a microfluidic electrolyzer cell (A = 1 cm^2 electrolyzer area), based on the work of the Kenis Research group. This consisted of two stainless steel electrodes with 2 mm deep gas chambers, and gas inlet and outlet ports formed in them. A 2 mm thick PMMA separator with a 0.5 cm × 2 cm sized window and Ø = 1 mm electrolyte solution inlet and outlet was used to separate the anode and cathode electrodes. Six bolt screws were used to assemble the cell, gradually applying a torque of 1.5 Nm.

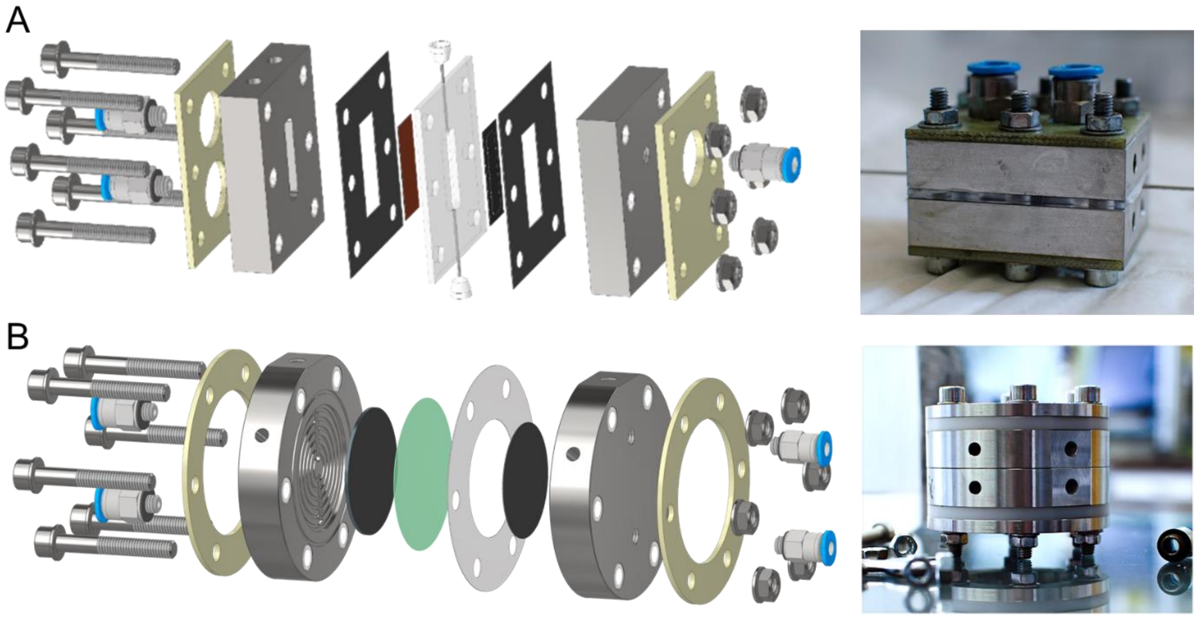

Figure S2. Schematic exploded and photographed assembled (A) microfluidic and (B) zero-gap electrolyzer cells.

Effect of the cell voltage on the rate and selectivity of electrochemical CO_2 reduction

We tested the influence of the applied cell voltage under the conditions later used for the long-term electrolysis experiments. The partial current density for CO production gradually increased with the cell voltage and resulted high product selectivity.

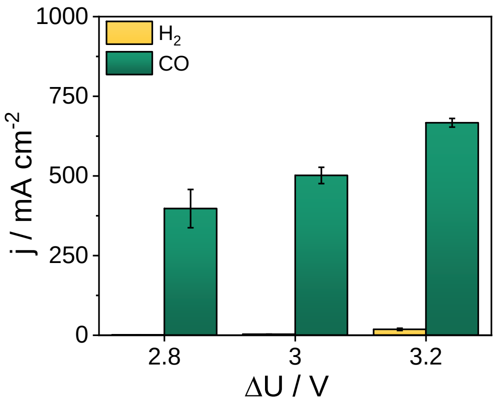

Figure S3. Bar diagrams for H_2 and CO partial current densities. Measured in the zero gap electrolyzer cell at T = 60 °C 0.05 M CsOH anolyte and 12.5 cm^3 cm^{-2} min^{-1} CO_2 feed.

Long-term electrolysis in zero-gap CO_2 electrolyzer cells under dynamic power load conditions

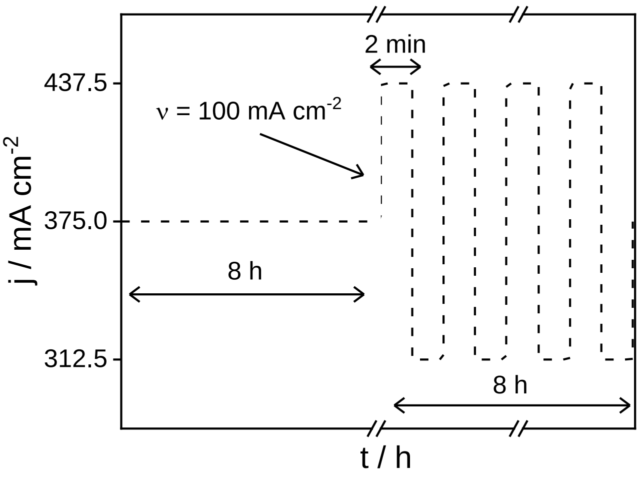

Figure S4. The current profile that was applied repeatedly during the measurements shown in Fig. 1A in the manuscript

Long-term electrolysis in zero-gap CO_2 electrolyzer cells at constant current

We performed galvanostatic measurements to assess the long-term stability and efficiency of CO_2 electrolyzers. Here we show examples where over 100 h electrolysis was performed in the zero-gap electrolyzer cell at different current densities.

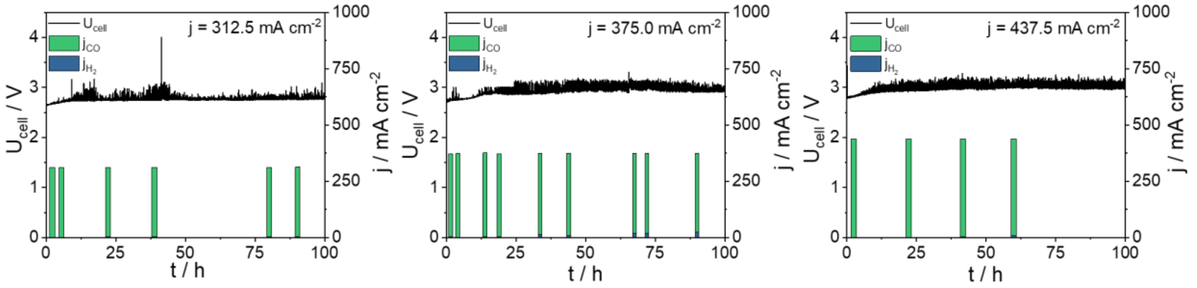

Figure S5. Total cell voltage and partial current densities for CO and H_2 production during constant current electrolysis (j = 312.5, 375, and 437.5 mA cm^{-2}, T_{cell} = 60 °C, 0.05 M $CsHCO_3$ anolyte, 12.5 cm^3 cm^{-2} min^{-1} CO_2 feed).

Long-term electrolysis in zero-gap CO_2 electrolyzer cells under dynamic power load conditions, mimicking the power output of a PV cell

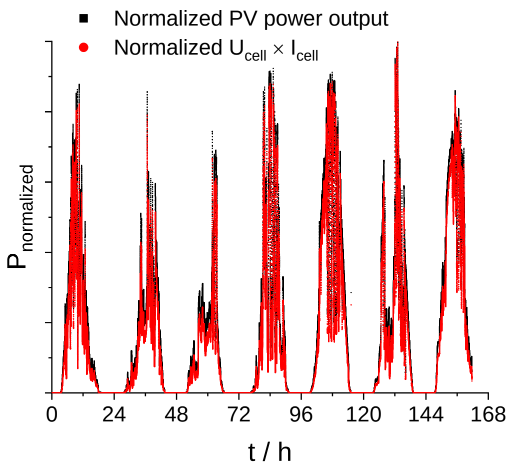

Figure S6. Comparison of the normalized PV power output and the cell power load during the measurement shown in Fig. 1D, with controlling the cell current according to the profile of the PV power output.

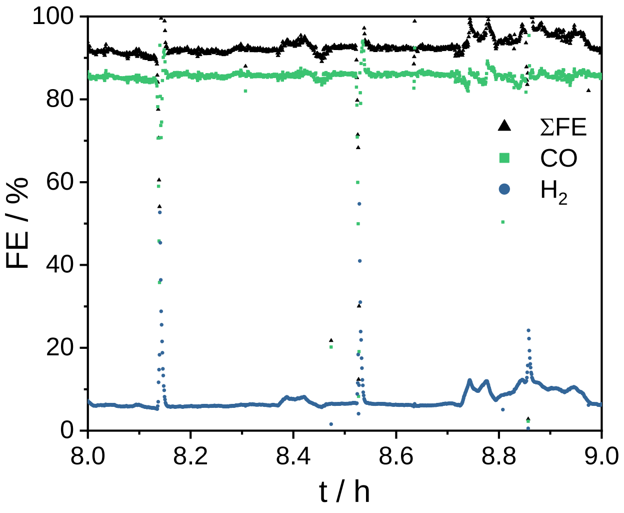

Figure S7. Faradaic efficiency of CO and H_2 formation and the sum of these (ΣFE) during the experiment are shown in Fig. 1B-C in the manuscript.

Dynamic power-load operation of a microfluidic CO_2 electrolyzer cell

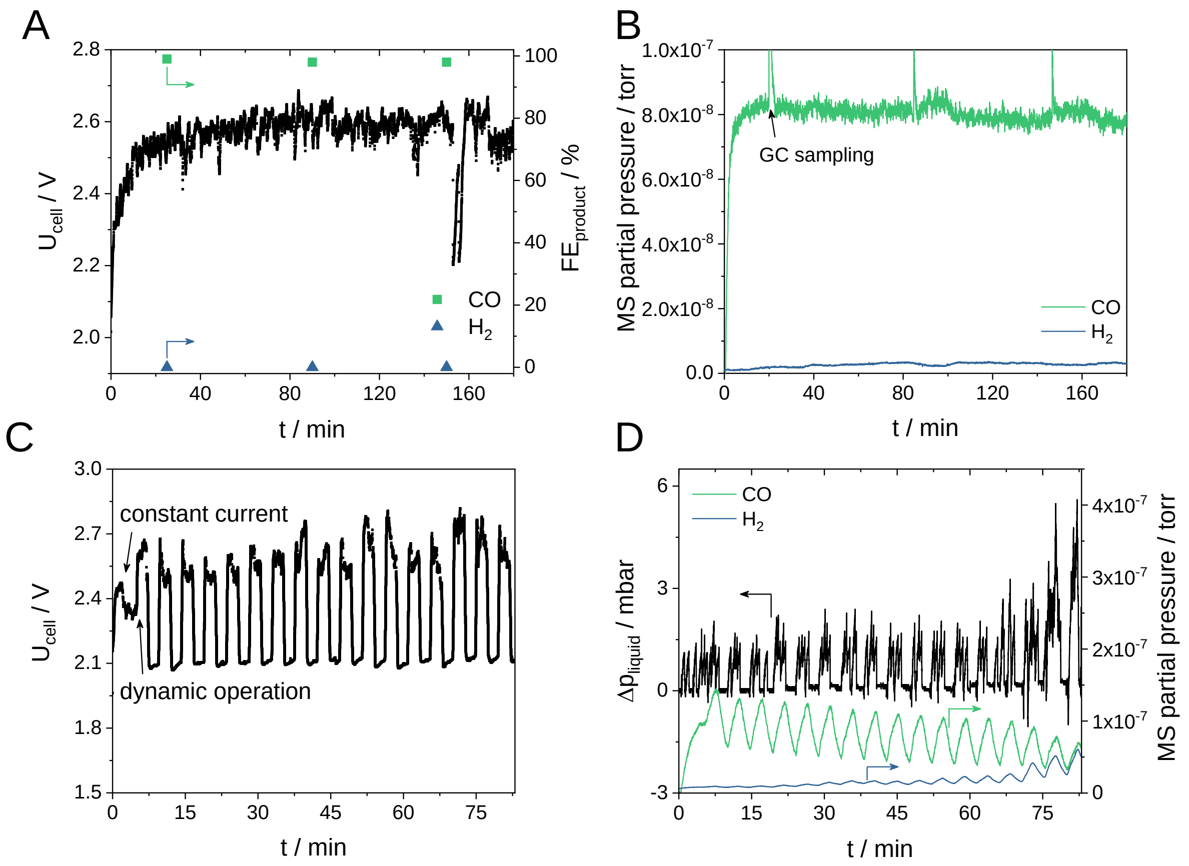

Figure S8. (A) Cell voltage and product distribution during electrolysis at j = 100 mA cm^{-2} current density. (B) Product distribution in the gas outlet stream sampled by an m/z analyzer during the electrochemical protocol presented in (A). (C) Cell voltage during electrolysis applying dynamic conditions. The current density was maintained at 100 mA cm^{-2} for 5 minutes. In the following, the current density was alternated between 50 and 150 mA cm^{-2} with 2 min periods (current density was changed in between holds applying a ramp with 5 mA cm^{-2} sweep rate) until flooding of the cathode GDL was observed. (D) Pressure of the liquid stream and product distribution in the gas outlet stream recorded during the electrochemical protocol presented in (C). Experimental conditions for all experiments were: 1 M CsOH anolyte fed to the cell without recirculation at a rate of 0.5 cm^3 min^{-1}, 20 cm^3 cm^{-2} min^{-1} cathodic CO_2 feed, room temperature.

As a first step, the operation of the microfluidic CO_2 electrolyzer cell was monitored at a constant current density of 100 mA cm^{-2} (Figure S8 A and B). Both the cell voltage and the Faradaic efficiency of the formed CO remained stable throughout the three-hour experiment. To see if similar conclusions can be made if the cell is operated under dynamic load conditions,

the current density was alternated between 50 and 150 mA cm^{-2} with 2-minute periods after an initial hold at j = 100 mA cm^{-2} for 5 minutes. Since the GC protocol takes approximately 15 minutes to finish after each acquisition step, the amount of the formed CO and H_2 was in-situ monitored with an m/z analyzer during these experiments (Figure S8C and D). Clearly, the amount of CO closely follows the variation in the applied current density. At the beginning of the experiment, only trace amount of H_2 formed, but its amount gradually increased over the course of the experiment, in parallel with the partial and then total flooding of the cathode GDE.

Liquid and gas pressures during the dynamic experiments were monitored by adding pressure transmitters to the setup. One was placed in front of the cell in the cathode gas inlet monitoring pressure changes in the gas stream, while the other was placed in the liquid stream right after the cell, connected to the outlet tube via a T-connector giving insights on the pressure change in the electrolyte stream. Change in the pressure in the gas phase was only detected after the flooding of the cathode GDE because of the transmission of liquid in the cathode gas outlet partially blocking it (data not shown). Contrastingly, the pressure in the liquid phase scales with the applied current density (Figure S8D): when the cell was operated at high current density, a higher liquid pressure was measured due to the excess formation of gas bubbles at the electrode/electrolyte interface. We note that this data was collected outside the cell, meaning that the obtained *trends* depict the changes, but only qualitatively (due to the buffering effect of the volume of the tubing after the cell). However, since we were not able to measure the pressure difference inside the cell, the exact values shall be handled with caution.
